# Supplementary material for: Neglect of publication bias compromises meta-analyses of educational research
Source: PLoS One. 2021 Jun 3;16(6):e0252415. doi: 10.1371/journal.pone.0252415 (PMC8174709; doi:10.1371/journal.pone.0252415)
Supplement: Attachment — Submitted filename: Response to reviewers.docx [file pone.0252415.s001.docx]

Dr. Vance Berger,

Academic Editor

PLOS ONE

May 4^th^, 2021

Dear Dr. Berger,

Re: Manuscript reference ID: PONE-D-20-29585R1 titled “Neglect of publication bias compromises meta-analyses of educational research“.

Please accept our sincerest thanks for the opportunity to revise this manuscript. We deeply apologize that it took us so long to deliver the revision. We would also like to express gratitude to the reviewers for their precious time and willingness to read and comment on the manuscript. We believe that in the revised version of the manuscript, we were able to address every one of the concerns raised.

In the letter below, we state the reviewer’s point (indicated by frames), provide our response, and copy-paste the relevant revised or added part from the manuscript (indented, single-spaced). In case the text is too long, we indicate the line numbers where the revised/added part can be found.

**The Editor**

When submitting your revision, we need you to address these additional requirements.

1. Please ensure that your manuscript meets PLOS ONE's style requirements, including those for file naming. The PLOS ONE style templates can be found at

https://journals.plos.org/plosone/s/file?id=wjVg/PLOSOne_formatting_sample_main_body.pdf and

https://journals.plos.org/plosone/s/file?id=ba62/PLOSOne_formatting_sample_title_authors_affiliations.pdf

We have revised the manuscript to comply with the journal style requirements.

1) Please include additional information about the rationale for choosing these specific journals and whether they're representative of a larger number of publications.

Our apologies for not being more explicit about the target of our inference. In the revised version, we have tried to clearly outline why have we chosen to survey the most recent meta-analyses published in the two highest-profile educational journals specializing in systematic reviews.

Please see our elaborate response to the same point (Point #1) raised by the Reviewer #2, along with the list of revisions.

2) If you intend to provide a current overview of publication and reporting practices at these journals, please include relevant publications from the last year in your study.

Thank you for the suggestion! We have now included and reviewed the entire population of systematic reviews published in two flagship educational review journals during the last five years (from January 2016 to December 2020). Our sample size thus went from 62 to 110 included meta-analyses (292 systematic reviews were screened).

Thank you for stating the following in the Author Note Section of your manuscript:

[This work was funded by the Czech Science Foundation under grant no.

21 [P402/12/G130], by the Slovak Research and Development Agency under Grant no. [APVV-

22 17-0418 and APVV-18-0140] and by project PRIMUS/20/HUM/009.]

We note that you have provided funding information that is not currently declared in your Funding Statement. However, funding information should not appear in the Acknowledgments section or other areas of your manuscript. We will only publish funding information present in the Funding Statement section of the online submission form.

Please remove any funding-related text from the manuscript and let us know how you would like to update your Funding Statement. Currently, your Funding Statement reads as follows:

[The funders had no role in study design, data collection and analysis, decision to publish, or preparation of the manuscript.]

Please include your amended statements within your cover letter; we will change the online submission form on your behalf.

We apologize for that. We removed the funding statement from the manuscript and moved it to the cover letter.

I agree with Reviewer #1 that bias detection is quite important. In fact, a compelling case can be made that it is of paramount importance, and that any researchers would be negligent for NOT undertaking such an effort. This is what I argued in my 2005 book regarding selection bias specifically, but the sentiment applies equally fervently to ALL types of bias. So while we hope that you will address ALL reviewer comments, please pay special attention to this one. Thank you.

We concur that our recommendation regarding publication bias correction was poorly worded. Please see our response to the first point raised by the Reviewer #1.

**Reviewer #1**

This study investigates how publication bias was assessed in educational meta-analyses. It is generally well written, and the code and data are publicly available. Although I disagree with some viewpoints by the authors, I think this study presents some novel results to guide future educational meta-analyses. My detailed comments are as follows.

Page 2, lines 38-41 in the abstract (and several places in the discussion). The authors dislike the trim and fill method, and treat selection models as state-of-the-art methods. I understand that the trim and fill methods have been shown to have limitations in several settings, but I don’t think it is “outdated.” It is intuitive and can be easily implemented by many software programs; it is also currently widely used as shown in this manuscript’s results. The original authors of the trim and fill method suggested it as a way of sensitivity analyses, which could effectively inform whether the meta-result is sensitive to potential publication bias.

On the other hand, the authors recommend the use of selection models. Although I generally agree with this, like the trim and fill method, they also depend on specific assumptions, and the bias-corrected results may not be reliable if the assumptions are seriously violated in a meta-analysis. Also, many selection models exist; the question is which one should be trusted if the authors want to rely on the bias-corrected result.

We agree that calling the trim & fill method "outdated" was probably too confrontational and short-sighted. Indeed, we should have probably taken a more measured stance here. On the one hand, several recent simulation studies suggest that trim & fill fares poorly especially with respect to Type I error rates. For instance, across multiple conditions, trim & fill showed a mean Type I error rate of .613 in Stanley, Doucouliagos, and Ioannidis (2017), .637 in Alinaghi and Reed (2018), .518 in Bom and Rachinger (2019), and .655 in Carter, Schönbrodt, Gervais, and Hilgard (2019). Although it did not perform that badly in terms of mean squared error or 95% CI coverage, these simulation studies provided rather clear evidence that it consistently leads to upward bias (this is, as with some other methods, exacerbated as heterogeneity increases). Also, the widespread use, intuitiveness, or convenient availability, and the actual statistical performance under bias are independent considerations. On the other hand, we concur that it is still superior to reporting just the naive random-effects meta-analytic estimates. Also, there are situations where trim and fill might perform well so it shouldn't probably be dismissed offhand.

Regarding the selection model, we definitely agree that like any other statistical model, these models have their assumptions too. If they are violated or the model is misspecified (for instance, if the weights in the step function are defined by the researcher), it can lead to erroneous results.

Although we think that the recommendation "reliance solely on trim & fill is not enough" is still warranted, we revised our recommendation section so that we no longer recommend what not to use – but instead what to use. In contrast to the previous version, we now explicitly suggest trying to employ statistically principled methods – like selection models, which are based on maximum likelihood estimation.

Relatedly, we tried to provide more measured recommendations regarding the use of multiple-parameter selection models. Although discussing the statistical properties of these methods (in fact, a conceptually heterogeneous family of methods) is beyond the scope of the present paper, we have revised relevant parts to make more explicit, that the ability of even the selection models to effectively and reliably recover the underlying unbiased effect size is fairly limited. Please see the revised recommendation #3 on pages 15-17.

Regarding the use of other methods, we refrain from naming specific methods and, instead, recommend using methods that have been found to perform reasonably well in the given specific context (see page 16, bottom).

Page 7, line 151. The authors include the p-curve as a type of selection methods; not sure it is within the list of recommended methods. As far as I know, some people are against this method. See a relevant paper: https://doi.org/10.1371/journal.pone.0149144.

Yes, we agree on that point. P-curve has some utility in examining the evidential value in a given, specific set of significant results. The skew of the p-curve distribution can help diagnose whether selective reporting can be ruled out as the sole explanation for a set of statistically significant findings. Just like implied by the paper you cite, or even the authors of the p-curve themselves (Simonsohn, Nelson, & Simmons, 2014), p-curve is not intended (and should not be used) to make any inferences about any underlying population of studies, and thus its use in fairly limited. The previous version of the manuscript did not list p-curve among the recommended methods. Nor does the revision. However, in the previous version of the manuscript, we did recommend the p-uniform* method (van Aert & van Assen, 2018). After the input for Reviewer #3, we reconsidered that recommendation, as one-parameter selection models are nested within multiple-parameter selection models.

Again, please see the rather substantially edited recommendation #3 on p. 15-17.

Page 8, lines 171-174. I don’t think the authors should presume that meta-analysts searched for gray literature by default. In medical meta-analyses, researchers usually conduct literature search in PubMed etc. for peer-reviewed studies, but not in Google Scholar, arXiv, or medRxiv, primarily due to concerns about the quality of manuscripts without peer reviews.

We are sorry for being unclear on that point. In fact, we did not presume that the authors of the meta-analyses included unpublished studies in their sampling frame. What we intended to say was that the authors did not need to use any specific phrase in their description of the search strategy to denote that such a search was carried out. Instead, it was sufficient if their search strategy covered databases that index unpublished material by default (like Google Scholar). We have tried to make the relevant part more explicit and, hopefully, more clear.

"Next, we examined whether the authors made an effort to include grey literature, or relied exclusively on peer-reviewed journals. Here, we coded 1 if (a) searching for grey literature was mentioned explicitly, (b) if the search strategy covered at least one database in search strategy that indexes unpublished material (e.g., preprint servers, Google Scholar, or ProQuest Dissertations), or (c) if the authors requested unpublished studies from professional LISTSERVs. We coded 0 only if the authors explicitly stated that unpublished status was an exclusion criterion.”

Page 14, line 340. I strongly disagree with “Bias detection is pointless, always try to correct for publication bias.” Bias detection indicates whether the meta-result is potentially subject to bias or not; it is of course meaningful because decision makers can use it to assess the reliability of the synthesized evidence. It also gives the direction of the potential bias. If the bias direction is toward the null, decision makers could rate down the evidence; if all the meta-result is nearly unchanged in all sensitivity analyses, it could be treated as reliable evidence.

We agree that this recommendation was very poorly articulated and even misleading. Of course, we fully agree with your description of the merits of bias detection. As was also pointed out by the editor, detection of all types of bias (including publication bias) is of paramount importance in appraising the quality of the empirical evidence. Our point was that the outcome of the bias detection should not guide the decision whether to try to adjust for bias or not.

In the revision, we deleted some misleading statements like:

"The question then is, does bias detection help in advancing educational research? Probably not, as it is not asking a useful question."

"Bias detection is pointless.."

At the same time, we have revised relevant recommendations as follows.

"2. *Try to correct for bias even if none was detected*. Detection of all types of bias has important diagnostic value in appraising the quality of evidence. Informal assessment (eyeballing the funnel plot; see [50, 53]) or testing for the presence of publication bias should, however, not guide the decision whether to adjust or not. Publication bias detection tests have been shown to lack statistical power in many settings (see [41]) and in the case of publication bias, they address a hypothesis that is known to be usually false [40]. Authors and editors alike are known to disfavor negative results [54, 55]. These then represent a tiny fraction of published evidence in social sciences [56]. It is simply not the case that all studies (and effects within those) get published regardless of their results. Such selection always leads to bias. Correction for publication bias should thus practically always be an integral part of the meta-analytic workflow."

"Publication bias is, in fact, a property of the research process, not individual studies. Any product of that process – whether or not it shows evidence of it – will thus be biased [40]. Because sampling a seemingly unbiased set of studies does not make the underlying process unbiased, one should make effort to correct for bias by default. The decision to correct for bias should not be contingent upon the outcome of bias detection. The median number of included effects in meta-analytic models was only *k* = 15. If the number of studies, and thus the power for detecting other than a pronounced pattern of bias is low [14, 41], a null outcome from a bias detection test may provide a false sense of confidence."

Moreover, it is not feasible to “always try to correct for publication bias” in many meta-analyses. For example, in medical meta-analyses, the number of included studies is usually quite small, say less than 10 or even 5 studies. In such cases, it is a big statistical change to estimate the complicated selection models; some selection models could even have much more parameters to be estimated than the available studies. In addition, as I mentioned earlier, all selection models depend on specific assumptions, while the true publication bias scenarios vary greatly across meta-analyses. It is also impossible to strictly check those assumptions. There are many selection models, and they likely produce very different bias-corrected results for the same meta-analysis; which model should be trusted? In my view, all statistical methods (including the trim and fill method and the selection methods) may serve as sensitivity analyses; the bias-corrected results should be interpreted on a case-by-case basis by evaluating if the assumptions made in the statistical methods are reasonable for a given meta-analysis.

This is a closely related point to the one above and, again, we cannot but agree. Although we argue that meta-analysts should *try* to adjust for publication bias by default, there are situations in which bias correction would not lead to informative estimates. A low number of included independent effects is definitely one of them. In that case, the only sound conclusion may be that the given research program is not yet ripe for a sufficiently informative synthesis.

We have reordered and revised the relevant parts so that the following comes right after the recommendation that "[c]orrection for publication bias should thus practically always be an integral part of a meta-analytic workflow":

“That said, bias correction usually requires substantively larger number of included effects to provide an informative estimate. If the number of included independent effects is too small (may also be due to clustering of effects), no proper adjustment may be viable and publication bias needs to be tacked by simpler sensitivity analyses (e.g., [47]). Put simply, sound evidence synthesis of a given research program requires a fair amount of cumulative empirical evidence. Sometimes, the only sound conclusion is that the given research program is not yet ripe for an informative synthesis"

Regarding the problem of choosing among the many existing selection models, we recommended employing a model averaging approach, which is a very general, principled approach to the issue of model misspecification. Here is the revised part on the selection models:

"Instead of relying on ad hoc methods with largely unknown statistical properties, it is advisable to base meta-analytic inferences on statistically principled correction methods, like the multiple-parameter selection models. The advantage over many other bias correction methods is that this highly flexible family of models attempts to model the functional form of the biasing selection process directly [see 7]. On the other hand, different implementations of selection models impose different assumptions and have different shortcomings [7]. One of the most general solutions to such an issue is then to average over various models to make bias correction more robust to model misspecification [57, 58]. For selection models, this is now easily done in *R* or in statistical package *JASP* [57]. For a different Bayesian implementation of selection models that allows to model not only publication bias but also p-hacking [59]. In situations where the number of included effects is too small, selection models can even be used as a sensitivity analysis. In the Vevea & Woods approach [60], one can fit a series of fine-grained step function models where the selection weights are not estimated but defined a prori. In doing so, the model gives up the ability to provide a single best guess of unbiased effect size. On the other hand, it makes it possible to examine the results assuming different forms and severity of bias (e.g., moderate, severe, and extreme selection) [60, 7]."

In the results, it would be helpful to report the information about the number of studies included in the 175 educational meta-analyses. The number of studies is an important factor that affects the statistical powers of statistical tests for publication bias and the uncertainties of the bias-corrected results.

We have coded this information and report the results in the manuscript. The median number of included effects across all the reported meta-analytic models (excluding meta-regressions) drawn from included meta-analyses was k = 15.

For coding the number of included effects in meta-analytic models k, we used tables, forest plots, and searched for the information in the text (in that order). If the number of effects were not reported directly, we tried to estimate k based on degrees of freedom for the Q test. When it was not clear whether, e.g., the authors used hierarchical models, or averaged the dependent effect sizes, we applied the benefit of the doubt principle and coded the larger number.

Minor comments

Page 6, line 131. Both (1) and (2), or (1) or (2)? I thought that a meta-analysis must present a quantitative synthesis of primary studies; otherwise, it could be only called a systematic review.

We apologize for being unclear. The two inclusion criteria for the present meta-study were the following: (1) the paper had to include a *meta-analysis* (2) providing a quantitative synthesis of *primary studies*. The essential piece of information in the second point was not "quantitative synthesis" but "quantitative synthesis of primary studies". The aim of this criterion was to exclude the umbrella reviews which synthesize the results of multiple meta-analyses. We have revised the given part to make the point clear as follows:

"The only inclusion criterion was the following: the paper had to include a *meta-analysis* providing a quantitative synthesis of *primary studies*. No restrictions on the type of synthesized effects were applied. We excluded meta-research studies on methodological aspects or umbrella reviews (reviews of meta-analyses)."

Page 6, line 135. The “;” should be “:”.

Thank you for noting that.

Page 6, line 137, “meets” -> “met”.

Corrected.

Page 8, lines 189-190, delete “Data for included meta-analyses are available at osf.io/ephba”; it is repeated (lines 183-184).

Deleted.

Page 8, line 191, “.” instead of “:” after “follows”.

Corrected.

Page 17, line 411, should the “.” before “Cautiously” removed?

Edited. We see now that the word "cautiously" was not functioning well in the given context.

Page 19, line 450, what does “only labels it as such” mean?

We tried to unpack the statement a bit to make the point more explicit.

"Pre-registration (e.g., on the Open Science Framework or a prospective register of systematic reviews like PROSPERO) does not preclude data exploration. It only marks a demarcation line between exploratory and confirmatory research"

**Reviewer #2**

The study presents a systematic review of meta-analysis in select journals that are considered flagship (the objective reason for this selection is not stated). Therefore the authors should not use the meta-analysis/study as the study design throughout the manuscript. A potential bias was introduced by not including all available literature on the subject and this should be stated as the limitation of the study. Furthermore, the Journal requires reports of systematic reviews and meta-analyses to include a completed PRISMA (Preferred Reporting Items for Systematic Reviews and Meta-Analyses) checklist and flow diagram to accompany the main text. Authors must also state in their “Methods” section whether a protocol exists for their systematic review, and if so, provide a copy of the protocol as supporting information.

Apologies for not being clearer and more explicit about the points you raised above. The present study is not a systematic review. Instead of sampling all published educational meta-analyses, we took a different approach. In our meta-study, we studied *all* systematic reviews published in two flagship educational review journals across the last 5 years. By intentionally selecting the most recent meta-analyses published in the two highest-impact review journals in education, we were trying to examine the practices of publication bias correction in arguably state-of-the-art meta-analyses that have the highest potential to steer practices and policies in education. The aim was thus to provide a steel man version of the assessment.

Although not a systematic review, we believe the present study provides an informative picture about the recent state-of-the-art meta-analyses. We see such a goal as legitimate – it is essentially the same approach that some other high-profile meta-studies have chosen. For instance, the Reproducibility Project: Psychology (Open Science Collaboration, 2015) sampled only from 3 high-ranked journals, Camerer et al. (2018) evaluated the replicability of social science experiments published exclusively in Nature and Science between 2010 and 2015, etc.

In multiple places of the revised manuscript, we are now trying to be very clear about the character of the present study, target population of studies as well as the limitation of such design.

The following was added/revised:

“In the present meta-study, we took the entire population of recent meta-analyses published in two flagship educational review journals (Review of Educational Research and Educational Research Review) and examined if and how they accounted for publication bias. Arguably, this population of studies should reflect the contemporary state-of-the-art in educational meta-analyses. The present study is therefore not a systematic review. By picking only the most recent meta-analyses from the two highest-impact specialized review journals in the field of education, we aimed to examine the population of state-of-the-art meta-analyses that have the highest potential to steer practices and policies in education. [1]"

"[1] The results of the study thus do not pertain to the entire population of meta-analyses in education. We assume, however, that meta-analyses published earlier or in lower-tier or non-specialized journals do not fare systematically better in terms of dealing with bias."

"The protocol was not pre-registered, as the present meta-study is not a systematic review and it tested no a priori hypotheses."

"The present meta-study examined how publication bias was dealt with in the population of meta-analyses reflecting the contemporary state-of-the-art in the field of education."

Please include the title Introduction as required by the Journal.

We are sorry for the omission. Section title added.

Please define abbreviations upon first appearance in the text (e.g. pg. 3 randomized control trials RCTs).

We went through the manuscript and defined all abbreviations.

Consider shortening the text on pg.5. It is not imperative to state every field the publication bias has been studied. A simple citation would suffice [10,11,17-29].

Thank you for the suggestion. Edited as suggested.

Please rewrite the second sentence in the Coding paragraph of the Methods section. I believe a part of the sentence is missing.

Indeed, the syntactic structure was not good. Our apologies for being unclear. We revised the relevant part as follows (with the preceding sentence for context).

"For each meta-analysis, we manually screened the method and results sections and coded whether the authors used methods for detection and correction of publication bias and reported the results. That did not include the risk of bias assessment at the level of individual studies (evaluation of possible methodological confounding effects related to randomization, blinding, etc.).”

Figure 1. The squares visualization is not informative enough to get a sense of proportions - bar plots would be better. This article could be informative: Sperandei S. The pits and falls of graphical presentation. Biochem Med (Zagreb). 2014;24:311-320

We concur that a barplot has a better data-ink ratio. We have replaced the waffle plot with a single percent stacked barplot.

Figure 2. The explanation on y-axis is missing. The figure should be self-explanatory.

Apologies for that. We have added the y-axis label and retouched the layout of the plot a bit.

On pg. 11, 1st paragraph - "The question then is... Probably not.." Actually, this is not the question of this study, so please omit these two sentences.

The given part was omitted.

In the discussion the authors focus more on giving the recommendations how to deal with the publication bias in general which is not reflected in the title of the study. The results of the study seem merely an afterthought, particularly concerning that none of the included meta-analysis adjusted their conclusions based on the detected bias. The authors should discuss their results and how this potentially affects the field of educational research. Avoid repeating the results (numerically) and instead focus on the interpretation of the data.

We have substantially revised the entire Discussion section. We have shortened unnecessary descriptions of the results, reordered a lot of content, and put more focus on what the implication of the results are. After including and coding more recent meta-analyses, we identified a few meta-analyses that explicitly based their conclusions about the magnitude of the target effects on the adjusted estimates. However, given that such practice is still rare, we see merits to focus on discussing the why and how of addressing the publication bias in educational meta-analyses. We believe that most of our recommendations are applicable not just in syntheses of educational research, but also in many other fields wrestling with the issue of publication bias.

That said, your comment pushed us to provide a more balanced discussion about the results of our study, implications of publication bias for the field of education, and what can be done about it. We feel that this made the discussion section more concise but also more informative for meta-analysts in the field of education. Should you feel that we're not there yet, we will gladly follow your advice.

The recommendations (elaborations) could be concised for better impact.

Please see our response to your previous suggestion. We have tried to cut down on non-essential or repetitive stuff and also removed a substantial portion of meta-commentary. Of course, we followed the suggestions of other reviewers for some additions too, but we think that the discussion section is relatively well informative and it reads better now. Although our aim is to provide the reader with all the information and nuance needed for a sound treatment of publication bias (at least as see it), if you still think that we should cut down on length even more, we will try to do so.

The conclusion is supported by the results in the text and the abstract, however, the authors again mostly focus on the general problem of publication bias. Considering the study focused on educational research so should be the conclusion.

In the revision, we tried to make sure the conclusions explicitly apply to educational research. Please see also our response to the point you made two paragraphs above.

The manuscript is written in standard English, however, I am not a native English speaker. Perhaps some sentences could be shortened for easier reading flow, but I am leaving this to authors' discretion.

We went through the manuscript, split most long, run-on sentences, and edited the text to improve readability.

**Reviewer #3**

The paper discusses (neglect of) publication bias in meta-analysis in educational research. The paper in large part consists of two parts. First, there is a review of meta-analyses in Review of Educational Research and Educational Research Review. Second, there is a set of recommendations. The former seemed well-conducted to me. However, the recommendations did not necessarily always follow from the empirics or prior literature from what I could tell.

More focused comments follow:

1. Page 1: While it is true that “the full body of published or otherwise available studies represents a biased sample of the conducted studies,” it is also biased relative to the hypothetical population of “all conceivable studies.” It is hard to know what the right population we should seek unbiased samples from, and this perhaps warrants elaboration from the authors.

Being it early in the manuscript, we did not go into more nuance, but we discuss that point in the Discussion.

"Apart from the difficulty to recover the true underlying effect size, the meaning of an average effect cannot be detached from research designs and operationalizations of the included studies. These are, in fact, a non-random sample from a population of all relevant studies that could have been conducted [48]. Meta-analytic estimates are thus not comparable if the effect-size-relevant design features are not uniformly distributed or controlled for (see [49]). All that makes it difficult to assess (1) whether the results of a meta-analysis are robust to bias, (2) to what universe of studies do the results generalize if that is the goal, and (3) what is the theoretical relevance of the meta-analytic average effect sizes (see [48, 49]).”

If you think that we should go deeper into that issue, we will gladly do so. We also very briefly make that point in the sentence you referenced:

"As a matter of fact, the full body of published or otherwise available studies represents a biased sample of the conducted (and all conceivable) studies."

2. Meta-analysis has traditionally been used for the estimation of effect sizes, in particular the variation in effect sizes and moderators of this variation—not for providing a p-value about the existence of an effect let alone a declaration of “statistical significance” based on some p-value threshold being exceeded. I think the paper should be revised to shift away from discussion of things like false positives and false negatives and more toward effect sizes. This is consonant with how meta-analyses have traditionally been used and with recent call to abandon / retire statistical significance.

Throughout the revised manuscript, we have shifted away from the NHST perspective and instead of Type I and Type II errors, we focus more on aspects like bias and precision in estimating the meta-analytic effect sizes.

3. The front end contained a lot of jargon briefly trotted out, e.g., allusions to Deborah Mayo’s concept of a severe test and related things which have not caught on within the mainstream statistical community let alone in her home discipline of philosophy. If you want to engage with her work, that is fine but I think you need to pursue that seriously rather than blithely in a sentence as here. The same holds for other jargony things in the paper.

We agree that loose use of jargon may cause confusion and is probably not productive. We went through the manuscript and removed such jargony content and ambivalent phrases.

4. Page 10: You note that “one meta-analysis employed the p-curve method but only to test for evidential value, without calculating a bias-corrected estimate.” In this case, Figure 2 should be corrected so p-curve is in light blue not dark blue.

This changed by including all meta-analyses published in the two target journals until 2020 (as requested by the editor). By including 48 recent meta-analyses, we were able to identify some that used selection models to provide bias-adjusted estimates. Accordingly, we have updated the figures and also retouched their layout.

5. Page 10: In addition to “testing for evidential value” and providing bias-corrected estimates, the p-curve also estimates so-called average power. It may be worth discussing this, especially in light of this recently-published manuscript:

McShane, B.B., Böckenholt, U., and Hansen, K.T. (2020), “Average Power: A Cautionary Note.” Advances in Methods and Practices in Psychological Science, 3(2), 185-199.

In the revised manuscript, we have taken a different approach and no longer discuss, nor recommend the use of the p-curve method. Please see our more detailed response to your comment #12. Should you think that discussing the issue of average power derived from meta-analytic methods like the p-curve would still function well in this manuscript, we will gladly do so.

6. Page 11: You make really nice points in the paragraph on the top of this page. However, beginning here and continuing through the rest of the manuscript, I think are too optimistic about bias-correction methods as if they can provide definitive adjustments. I think, as per your reference [7], you should urge caution in the use of these bias-correction methods. That is, they should be used less for obtaining a single estimate that purports to adjust for publication bias ex post and more for sensitivity analysis— that is, exploring the range of estimates that result from assuming different forms of and severity of publication bias.

As a matter of fact, we don't favor labeling publication bias adjustment as a "sensitivity analysis". But that is not because we think that some publication bias correction method can reliably recover true underlying effect sizes. None of the current methods can do that and we doubt that there will ever be such a method. We agree that publication bias adjustment should be employed *exactly* the way you describe. However, we think that labeling it as a "sensitivity analysis" gives too much weight to naive random-effects meta-analytic estimates and implies that the issue of tackling publication bias can be considered a sort of an afterthought. At least this is what we observe in the applied literature.

As we say, we fully agree with the point you make and have made numerous edits to do text to be clear about the limitations of any current statistical adjustment methods under bias, including the selection models.

Please see the following:

Recommendation #3, lines 348 – 385

lines 275 – 285

lines 286 – 296

lines 302 – 309

Minor edits were done practically in the entire discussion section.

7. Page 11: You write “Still, none of them explicitly aligned their main conclusions with adjusted estimates (as judged from abstracts and discussion sections), rendering the adjustment inconsequential.” Would you please say more about this? Another few sentences would help.

What we found was that less than one in ten surveyed meta-analyses that adjusted for the detected bias explicitly aligned their headline quantitative interpretations in the abstract or discussion sections with the results of bias-correction methods. The sentence you refer to was removed as it was redundant. We now report the details in the Method section.

"In case that the authors reported detecting publication bias and calculated an adjusted estimate, we coded whether they explicitly informed any of their substantive conclusions by adjusted estimates, that is, whether the correction was consequential for the interpretation of the synthesized data. This was judged by identifying the main verbal interpretation of the focal effect size estimates and the associated inference in the abstract and discussion and comparing those with the results of reported bias correction."

8. Page 12: You write “Currently, no bias correction technique can accommodate selective reporting at the study level (p-hacking, [44]) and publication bias simultaneously.” This seems wrong: selective reporting = publication bias = file drawer. In the other hand, p-hacking (a term which I do not like*) involves not selective reporting but multiple potential comparisons issues.

* https://statmodeling.stat.columbia.edu/2017/05/10/p-hacking-intention-cheat-effect/

As the notion of multiple comparison issue as the garden of forking paths does not imply an active pursuit of a significant result, we agree it is a broader notion that does not imply cheating. Sorry if we did not fully get your point – but we still see selective reporting (as multiple comparisons issue) and publication bias as distinct (but interrelated) phenomena. We see publication bias as type of bias where non-significant or negative focal findings are less likely to get written up and be published. On the other hand, there may be situations in which the researcher transforms a less publishable result into a result that has a higher chance of getting published. It may well be an honest desire for making a discovery. Thanks to a set of data-contingent decisions, (implicitly) selecting from a universe of possible analyses a favorable one, the given result is then less likely to be missing from the literature. As Friese & Frankenbach (2020) have shown, if selection for significance happens at the level of focal results (publication bias) as well as at the level of analytic pathways leading to those results (the issue of multiple possible comparisons), these two mechanisms interact, with the latter amplifying the effect of the former. That holds especially under moderate publication bias and small true effects. This is what we were trying to say on that point. We tried to revise the concerned section to make our point regarding the mentioned "selective reporting" clearer. If we are still missing the point, we would be glad to stand corrected.

“Currently, none of the widely used correction techniques can simultaneously accommodate selection for significance at the level of focal results (publication bias) as well as at the level of analytic pathways leading to those results (the issue of multiple potential comparisons, [44]).[4] Lastly, there is considerable inherent uncertainty associated with the adjusted effect size estimates [14, 15, 37, 46], also leading to lower statistical power for detecting more subtle effect sizes.”

[4] In this respect, Friese and Frankenbach have shown that the effect of publication bias gets amplified if some of the null or negative results are transformed into positive results – especially under moderate publication bias and small true effects [45].

9. Page 12: You write “If there is bias in the literature, it has been well demonstrated that unadjusted meta-analysis tends to detect an effect even if there is none, and the current adjustments are imperfect.” Again, please see my comment #2 and move away from this false dichotomy view of the world.

Edited. Please see our response to your comment #2.

10. Page 14, Recommendation 1: I repeat my comment #6 regarding optimism and sensitivity.

Edited. Please see our response to your comment #1.

11. Page 14: You write

“[I]n some analytical contexts, the goal may be just to summarize the results of a finite set of studies without attempting to draw generalizable inferences about unobserved studies (using fixed-effects meta-analytic models). Other than that, the assumption of every random-effects meta-analytic model that all the conducted empirical studies are (1) written up and (2) published regardless of the outcome is untenable in any field of social or behavioral science.”

It is simply not the case that use of random effects models implies some population of studies. This is one way to view the random effects model. However, it is also equally valid to say that the goal is “just to summarize the results of a finite set of studies” and use the random effects model as a means of providing principled shrinkage / regularization via the hierarchical model. Please correct this content.

Thank you for pointing that out. We have revised the given part as follows.

"Also, in some analytical contexts, the goal may be just to summarize the results of a finite set of studies without attempting to draw generalizable inferences about unobserved studies (using fixed-effects meta-analytic models).[1] If, on the other hand, the aim is to draw inferences about some underlying population of studies, the random-effects meta-analytic models impose the assumption that all the conducted empirical studies are written up and published *regardless of the outcome*. This assumption is untenable in most fields of social or behavioral science. All bias correction methods also make assumptions that may be problematic in certain contexts and none of them recovers the underlying effect size in a sufficiently reliable way."

[1] The random-effects model can – as any other hierarchical model – then be employed to regularize the estimates.

12. Page 16: You write: “These models should be preferably supplemented by a different approach, like the regression-based PET-PEESE model [35] or another selection method, p-uniform*.” I would not recommend PET-PEESE as it is as ad hoc method with no known statistical properties. I also would not recommend one parameter selection models like p-curve and p-uniform because they make unrealistic and untenable assumptions and because more general selection models like the three parameters ones and the other Hedges and Vevea ones nest the one parameter models (i.e., in the event the one parameter selection models is correct, the three parameter selection model will be able to detect / fit that).

We have removed the recommendation to use such non-principled methods and recommend that meta-analyses should strive to correct for publication bias using statistically principled approaches like selection models, ideally combined with a model-averaging approach to tackle the issue of model misspecification. In low-k scenarios where the estimation of the step function is an issue, we recommend approaching selection models from a sensitivity analysis perspective, using the Vevea & Woods approach.

Here is the revised section on that point.

"Instead of relying on ad hoc methods with largely unknown statistical properties, it is advisable to base meta-analytic inferences on statistically principled correction methods, like the multiple-parameter selection models. The advantage over many other bias correction methods is that this highly flexible family of models attempts to model the functional form of the biasing selection process directly [see 7]. On the other hand, different implementations of selection models impose different assumptions and have different shortcomings [7]. One of the most general solutions to such an issue is then to average over various models to make bias correction more robust to model misspecification [57, 58]. For selection models, this is now easily done in *R* or in statistical package *JASP* [57]. For a different Bayesian implementation of selection models that allows to model not only publication bias but also p-hacking [59]. In situations where the number of included effects is too small, selection models can even be used as a sensitivity analysis. In the Vevea & Woods approach [60], one can fit a series of fine-grained step function models where the selection weights are not estimated but defined a prori. In doing so, the model gives up the ability to provide a single best guess of unbiased effect size. On the other hand, it makes it possible to examine the results assuming different forms and severity of bias (e.g., moderate, severe, and extreme selection) [60, 7]."

We also tried to be very measured about the recommendation to supplement selection models with different approaches. Instead of recommending PET-PEESE or p-uniform*, we have taken a more general approach: applying distinct approaches and examining the degree of concord among the estimates has diagnostic value, as long as the employed methods have been shown to perform relatively well in similar scenarios as assumed in the given application.

"There are also several other approaches to publication bias correction [see 15] and new ones are continually being developed. In many practical applications, it may be sensible to supplement selection models with different approaches. By applying distinct methods, one can effectively explore how much variable are the effect size estimates under different assumptions about the selection process. However, interpreting the degree of concord between different methods assumes that the employed methods tend to perform well in the given analytic scenario. Correction methods can, in fact, disagree with each other not just due to different assumptions but also due to distinct biases [11].”

…

“In general, the choice of bias correction methods requires either making assumptions or having knowledge about a given research program, and should be preceded by a method performance check.”

…

“We recommend that, at least, when several adequate correction techniques indicate a sharp difference from the crude meta-analytic estimate, such estimate should lose much of its interpretational relevance, and the substantive inferences need to be informed primarily by the results of bias adjustments."

13. Page 18, Recommendation 7: It may be worth noting that the popular R meta-analysis package metafor now accommodates a wide variety of selection models via the selmodel function:

http://www.metafor-project.org/doku.php/news:news

Added. Thank you.

14. Page 19, Recommendation 9: I repeat my comment #6 regarding optimism and sensitivity. I will also note that it is unrealistic to ask for bias-correction attempts in all meta-analyses. Fair enough for simple two-level ones with a single identical dependent measure across the studies. However, when the meta-analysis has more than two levels or is multivariate or both, there are simply no methods extant to attempt bias-correction and it is not straightforward to adapt current methods to this setting.

We have revised the given section, removing some simplifying and redundant claims, as well as reiterating the words of caution.

Most of the relevant revised parts are copy-pasted in responses to previous points.

*Thank you all once again for taking your time to read and comment on this manuscript.*
